# Complete Genome Sequence of *Actinobacillus succinogenes* GXAS137, a Highly Efficient Producer of Succinic Acid

**DOI:** 10.1128/genomeA.01562-17

**Published:** 2018-02-22

**Authors:** Hongyan Zhang, Naikun Shen, Yan Qin, Jing Zhu, Yi Li, Jiafa Wu, Ming-Guo Jiang

**Affiliations:** a Guangxi Key Laboratory of Utilization of Microbial and Botanical Resources, Guangxi Key Laboratory Cultivation Base for Polysaccharide Materials and Their Modification, School of Marine Sciences and Biotechnology, Guangxi University for Nationalities, Nanning, Guangxi, China; b National Non-Grain Bioenergy Engineering Research Center, Guangxi Academy of Sciences, Nanning, Guangxi, China; c Biology Institute, Guangxi Academy of Sciences, Nanning, Guangxi, China

## Abstract

The bacterium *Actinobacillus succinogenes* GXAS137, an efficient producer of succinic acid, was isolated from bovine rumen in Nanning, Guangxi Province, China. Here, we present the 2.3-Mb genome assembly of this strain, which consists of 2,314,479 bp (G+C content of 44.89%) with a circular chromosome, 2,235 DNA coding sequences, 57 tRNAs, and 15 rRNAs.

## GENOME ANNOUNCEMENT

Succinic acid (SA) is an important C4-building chemical platform for many applications, including food, agriculture, and pharmaceutical (1, 2) production. Commercial SA is mainly produced through petrochemical processes, which bring environmental pollution and other concerns related to sustainable development (3, 4). As an alternative, SA can be manufactured using bio-based feedstock through microbial fermentation (5).

SA production has been accomplished by different microorganisms, including *Anaerobiospirillum succiniciproducens*, *Actinobacillus succinogenes*, *Basfia succiniciproducens*, *Mannheimia succiniciproducens* (6), *Saccharomyces cerevisiae* (7), and *Escherichia coli* (8). Although these organisms have achieved a competitive performance, *A. succinogenes*, a capnophilic anaerobic Gram-negative bacterium, is promising because of its ability to form SA naturally at appreciable yields and productivities from a broad range of carbon sources (9–11).

Recent study in our laboratory has isolated an efficient SA-producing strain, *A. succinogenes* GXAS137 (China Center for Type Culture Collection accession no. CCTCC M 2011399), which was originally isolated from bovine rumen in China. The strain can produce up to 95 g/liter of SA with different substrates, such as glucose, cane molasses, duckweed powder, cassava powder, and crude glycerol (12–14). To generate genomic insights into its SA production and relative gene regulation, we performed the whole-genome sequencing of *A. succinogenes* GXAS137.

The genome of *A. succinogenes* GXAS137 was sequenced at Beijing Novogene Bioinformatics Technology Co., Ltd., with massive parallel sequencing using Illumina technology. Two DNA libraries were constructed: a paired-end library with an insert size of 350 bp and a mate-pair library with an insert size of 6 kb. The 350-bp library was sequenced with an Illumina MiSeq and HiSeq 2500 platforms using a paired-end 300-bp strategy. A total of 700 Mb of filtered paired-end reads were obtained with SOAPdenovo software to reach a depth genome coverage of 200-fold (15). Gaps were closed by PCR and subsequent Sanger sequencing. Gene prediction was performed with GeneMarkS. The filtered reads were assembled with SOAPdenovo to generate scaffolds. Genome annotation was predicted with the NCBI Prokaryotic Genome Automatic Annotation Pipeline, and additional software was used to predict the other elements in the genome. tRNAs were predicted with tRNAscan-SE, rRNAs were predicted with rRNAmmer, and small RNAs (sRNAs) were predicted by a BLAST search against the Rfam database. PHAST was used for prophage prediction, and CRISPRFinder was used to identify clustered regularly interspaced short palindromic repeats (16).

The genome of *A. succinogenes* GXAS137 was characterized by a circular chromosome of 2,312,173 bp with a 44.89% G+C content without plasmids. The chromosome contains approximately 2,454 predicted genes, 2,235 protein-coding genes, 139 pseudogenes, 57 tRNAs, 15 rRNAs, and 8 sRNAs.

So far, only one complete sequencing genome of the *A. succinogenes* 130Z has been published and analyzed in detail (17). The availability of the complete genome sequence of strain GXAS137 not only will contribute to enriching the genome database but will also give us the opportunity to investigate further the genes related to the biosynthesis of SA.

### Accession number(s).

This whole-genome shotgun project has been deposited at DDBJ/ENA/GenBank under the accession no. NHRD00000000. The version described in this paper is the first version, NHRD01000000.
